# Preoperative assessment of bone density for dental implantation: a comparative study of three different ROI methods

**DOI:** 10.1186/s13005-024-00434-0

**Published:** 2024-05-17

**Authors:** Shiuan-Hui Wang, Lih-Jyh Fuh, Michael Y. C. Chen, Ming-Tzu Tsai, Heng-Li Huang, Shin-Lei Peng, Jui-Ting Hsu

**Affiliations:** 1 School of Dentistry, China Medical University, Taichung, 404 Taiwan; 2 Department of Dentistry, China Medical University Hospital, Taichung, 404 Taiwan; 3 Department of Biomedical Engineering, Hungkuang University, Taichung, 433 Taiwan; 4 Department of Biomedical Engineering, China Medical University, 91 Hsueh-Shih Road, Taichung, 40402 Taiwan; 5 Department of Bioinformatics and Medical Engineering, Asia University, Taichung, 413 Taiwan; 6 Department of Biomedical Imaging and Radiological Science, China Medical University, Taichung 91 Hsueh-Shih Road, Taichung, 40402 Taiwan

**Keywords:** Jawbone, ROI method, Cancellous bone density, Dental cone beam computed tomography, Dental implant site

## Abstract

**Background:**

Dental cone beam computed tomography (CBCT) is commonly used to evaluate cancellous bone density before dental implant surgery. However, to our knowledge, no measurement approach has been standardized yet. This study aimed to evaluate the relationship between three different regions of interest (ROI) methods on cancellous bone density at the dental implant site using dental CBCT images.

**Methods:**

Patients’ dental CBCT images (*n* = 300) obtained before dental implant surgery were processed using Mimics (Materialise, Leuven, Belgium). At the potential implant sites, the rectangle, cylinder, and surrounding cylinder ROI methods were used to measure bone density. Repeated measures one-way analysis of variance was performed to compare the three ROI methods in terms of measurement results. Pearson correlation analysis was performed to identify the likely pair-wise correlations between the three ROI methods.

**Results:**

The density value obtained using the surrounding cylinder approach (grayscale value [GV],523.56 ± 228.03) was significantly higher than the values obtained using the rectangle (GV, 497.04 ± 236.69) and cylinder (GV,493 ± 231.19) ROI methods in terms of results. Furthermore, significant correlations were noted between the ROI methods (*r* > 0.965; *p* < 0.001).

**Conclusions:**

The density measured using the surrounding cylinder method was the highest. The choice of method may not influence the trends of measurement results.

**Trial registration:**

This study was approved by the Institutional Review Board of China Medical University Hospital, No. CMUH111-REC3-205. Informed consent was waived by the Institutional Review Board of China Medical University Hospital, CMUH111-REC3-205, owing to the retrospective nature of the study.

## Introduction

Recently, the quality of medical care has garnered considerable attention from the general population. Because of the increase in the number of older individuals, concerns such as missing teeth and treatments to restore the normal occlusal function and aesthetically improve the region with missing teeth demand further studies [1]. With the development of dental, and medical techniques, dental implants have become the standard treatment option compared with the conventional fixed and removable dentures [2, 3]. Several factors influence the success of dental implant surgery [4–8]. One such important factor is postoperative osseointegration ability [4]. Osseointegration refers to the integration of a dental implant with the alveolar bone. Satisfactory osseointegration can improve the survival rate of dental implants [9]. If the bone quality at the dental implant site is satisfactory, the initial stability of the dental implant may be enhanced, and the satisfactory osseointegration ability may increase the success rate of dental implant surgery [10, 11]. Therefore, the bone quality and quantity at the dental implant site must be evaluated.

Bone quality can be evaluated through various approaches, such as sonography, stained sections, and dual-energy X-ray absorptiometry [12–14]. However, these approaches cannot measure the actual bone density and are not effective in routine clinical applications. Computed tomography (CT) is widely used [15–19]. Dental cone beam CT (CBCT) has also been adopted to measure the grayscale density (grayscale value [GV]) of images obtained for evaluating bone density before dental implant surgery [20–25]. The unit used for measuring bone density at dental implant sites through CT is Hounsfield unit (HU), whereas that used for measuring bone density through dental CBCT is GV [26, 27]. Both the values of CT and CBCT are associated with the attenuation coefficient of the scanned object, and the attenuation coefficient is associated with the actual density of the object [28, 29]Therefore, the site with higher radiographic density has higher bone mineral density.

Most studies using CT and dental CBCT to evaluate the bone mineral density at dental implant sites have assessed multiple regions of interest (ROI) [15–23, 25, 30]. In some studies, two-dimensional (2D) cross-sections have been used to measure bone density at dental implant sites within a rectangular area on a planar image [17, 18, 30]. By contrast, in some studies, a three-dimensional (3D) cylinder has been used to simulate the actual dental implant and measure bone density within the cylinder volume [19, 20, 23, 25]. Bone density in the area surrounding the cylinder has also been measured to study the area that will be in actual contact with the dental implant after dental implant surgery [15, 16, 21, 22]. Thus, the bone density at dental implant sites can be evaluated using different ROI methods. However, the ROI methods remain to be compared in terms of their results. Although multiple approaches have been adopted to measure bone density using CT/CBCT images, to the best of our knowledge, because of the inconsistency in measurement methods, it is impossible to determine the impact of ROI methods on jawbone density assessment using CBCT. Therefore, in the present study, we compared three ROI methods used for measuring cancellous bone density at dental implant sites in terms of their results. In addition, we investigated the likely differences and correlations between the ROI methods. Dental CBCT images were used for evaluation.

## Materials and methods

### Patient selection

This study was approved by the Institutional Review Board of China Medical University Hospital (approval number: CMUH111-REC3-205). Because of the retrospective nature of this study, the requirement of informed consent was waived. All procedures were conducted in accordance with the Declaration of Helsinki. Between August 2018 and March 2020, a total of 300 dental CBCT images were collected from 127 patients (men, 64; mean age of men, 53.9 ± 14.5 years; women, 63; mean age of women, 51.3 ± 15.3 years) who were indicated for dental implant surgery at the Dental Division of China Medical University Hospital were retrospectively selected. To participate in the study, individuals had to meet specific criteria, including being between the ages of 20 and 85 and having access to preoperative dental CBCT images. In addition, the exclusion criteria were the absence of metal implants (e.g., dental braces, bone screws, and bone plates) or motion artifacts in CBCT images.

The dental implant sites were the anterior maxilla [42], posterior maxilla (127), anterior mandible [39], and posterior mandible (107). In addition, the scanning parameters of dental CBCT (Promax 3D Max; Planmeca, Helsinki, Finland) were set: voxel size 200 μm, voltage 96 kV, and current 12.5 mA.

### Regions of interest methods

**Fig. 1:**
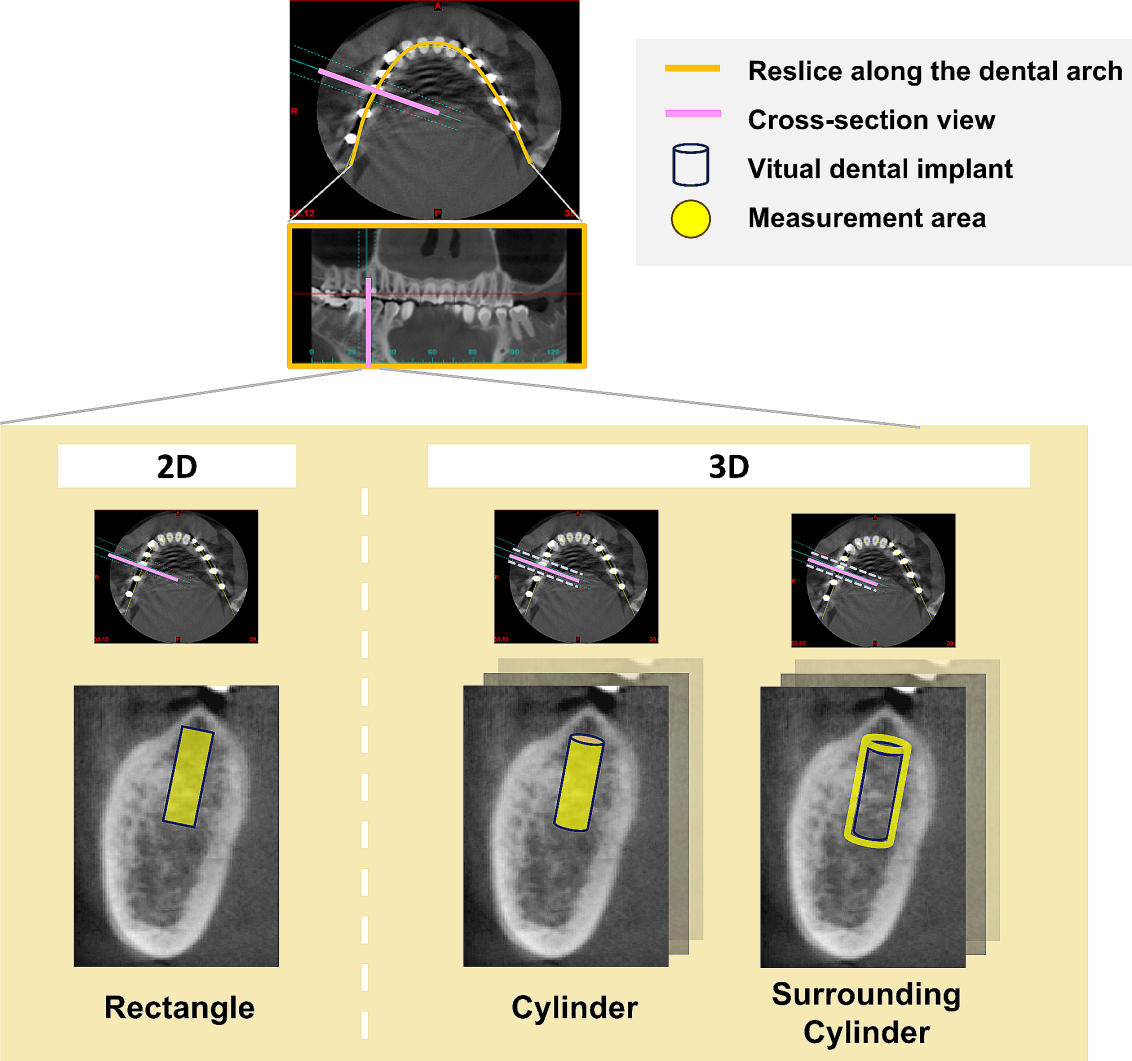
Three ROI methods used for bone density measurement: Rectangle, Cylinder, and Surrounding cylinder

The dental CBCT images were processed using Mimics (version 15.0; Materialise, Leuven, Belgium) according to the Digital Imaging and Communications in Medicine standard to estimate the density (GV) of the jawbone before dental implant surgery. The images were resectioned along the dental arch to obtain a plane orthogonal to the dental arch. Images were taken with the patients wearing surgical stents made by their dentists; the stent contained a radiopaque guide, which guided to the potential implant sites. The radiographic guide can help identify potential implant sites and determine implant angles in CBCT images. The radiographic density of the cancellous bone at the potential implant sites was measured, and the following three ROI methods were assessed in this study (Fig. 1). In addition, the present research conducted a scanning of the BMD calibration phantom (Micro CT-HA phantom, QRM GmbH, Moehrendorg, Germany) to facilitate the transfer of measurement values to the corresponding BMD values.

#### Rectangle (rectangular area in a single section)

In the cross-section image of the potential implant sites, the central slice of the radiographic guide was selected as the measurement slice. We selected the “measure density inside rectangle” function in Mimics to form a virtual rectangle (width, 3.5 mm; length, 11 mm) representing the dental implants. The mean density (GV) within the rectangle was measured. The BMD values were also recorded in g/cm^3^.

#### Cylinder (cylindrical area in a multislice section)

At the potential dental implant sites, we selected the “create cylinder” function (“analyze” section) in Mimics to form a virtual cylinder of the same size as the actual dental implant on a multislice section. The diameter and length of the cylinder were 3.5 and 11 mm, respectively. The mean density (GV) within the cylinder was measured. The BMD values were also recorded in g/cm^3^.

#### Surrounding cylinder (the area surrounding the cylinder in a multislice section)

We selected the “create cylinder” function in Mimics to form a virtual cylinder of the same size as the actual dental implant on a multislice section. The diameter and length of the cylinder were 3.5 and 11 mm, respectively. Subsequently, the “dilate” function (“morphology operation” section) in Mimics was selected to form a new cylinder by expanding the original cylinder outward by 1 pixel. Finally, the “Boolean operation” function was used to subtract the two cylinders to include the cancellous bone in the 1-pixel area outside the dental implant (the resolution of CBCT was set at 200 μm/pixel). The mean density (GV) in this area was measured. The BMD values were also recorded in g/cm^3^.

### Statistical analysis

Data are presented in terms of mean and standard deviation values. The Kolmogorov–Smirnov test was performed to analyze the normal distribution of the data obtained using the three ROI methods. For each method, one-way analysis of variance (ANOVA) was performed to compare jawbone regions (i.e., anterior maxilla, anterior mandible, posterior maxilla, and posterior mandible). Scheffe’s test was used for post hoc analysis. The three ROI methods were compared using repeated measures ANOVA. The correlations between the three methods were investigated using the Pearson correlation coefficient (r) test. All analyses were performed using SPSS (IBM Corporation, Armonk, NY, USA). Statistical significance was set at *p* < 0.05.

## Results

### Results of the normality test

The cancellous bone density values in CBCT (unit: GV) and BMD (unit: g/cm^3^) values obtained using the rectangle, cylinder, and surrounding cylinder ROI methods were 497.0 ± 236.7 GV (330.4 ± 108.33 g/cm^3^), 493.9 ± 231.2 GV (327.75 ± 103.64 g/cm^3^), and 523.6 ± 228.0 GV (53.09 ± 100.91 g/cm^3^), respectively. The Kolmogorov–Smirnov normality test revealed that the data showed a normal distribution (*p* > 0.05).

### Differences across jawbone regions

The data were further stratified by jawbone region and analyzed using ANOVA and Scheffe’s tests. No statistical difference was noted between the posterior mandible and anterior maxilla in terms of the estimated cancellous bone density; however, significant differences were observed between any other two regions (*p* < 0.05). Bone density was the highest at the anterior mandible, followed by the at anterior maxilla, posterior mandible, and posterior maxilla (Table 1).

**Table 1:** Measurement results stratified by jawbone region

ROI method	Anterior Maxilla	Posterior Maxilla	Anterior Mandible	Posterior Mandible
Rectangle	542.1^a*^	385.6^b^	723.4^c^	532.8^a^
Cylinder	541.9 ^a^	383.8 ^b^	710.2^c^	530.3^a^
Surrounding cylinder	581.5^a^	406.5^b^	744.2^c^	563.0^a^

*Results of the three regions of interest (ROI) methods (row); the same letter indicates no significant differences (*p* > 0.05)

### Correlations between the three regions of interest methods

Table 2 presents the Pearson correlation coefficients for the correlation analysis of the ROI methods, and the p-values for all measurements are less than 0.05. Significantly correlations were observed between the rectangle and cylinder methods (*r* = 0.992; *p* < 0.01), between the rectangle and surrounding cylinder methods (*r* = 0.965; *p* < 0.01), and between the cylinder and surrounding cylinder methods (*r* = 0.976; *p* < 0.01).

**Table 2:** Correlations between the three regions of interest methods

Pearson correlation coefficient (*r*)	All region (300)	Anterior Maxilla (40)	Posterior Maxilla (122)	Anterior Mandible (36)	Posterior Mandible (102)
#1 and #2	0.992	0.983	0.992	0.984	0.995
#1 and #3	0.965	0.946	0.961	0.940	0.962
#2 and #3	0.976	0.966	0.976	0.955	0.971

#1: rectangle; #2: cylinder; #3: surrounding cylinder

### Differences between the three regions of interest methods

Repeated measures ANOVA was performed to determine the differences between the three ROI methods in terms of results obtained for the same sample. Table 3 presents the results of repeated measures ANOVA. Except for the measurement results of the rectangle and cylinder methods in the four jawbone regions (*p* = 0.059) and those of the rectangle and surrounding cylinder methods in the anterior mandible region (*p* = 0.119), the remaining results indicated significant differences (*p* < 0.001). However, no significant difference was observed between the measurement results of the rectangle and cylinder methods in the four regions or between those of the rectangle and surrounding cylinder methods in the anterior mandible region. In the remaining regions, the measurement results indicated significant differences between various ROI methods.

**Table 3:** Differences between the three regions of interest methods in terms of measurements performed across jawbone regons

Repeated measures ANOVA	All region (300)	Anterior Maxilla (40)	Posterior Maxilla (122)	Anterior Mandible (36)	Posterior Mandible (102)
#1 vs. #2	0.059	0.977	0.433	0.059	0.267
#1 vs. #3	< 0.001	0.001	< 0.001	0.119	< 0.001
#2 vs. #3	< 0.001	< 0.001	< 0.001	0.005	< 0.001

#1: rectangle; #2: cylinder; #3: surrounding cylinder; vs.: versus

## Discussion

In this study, we compared three ROI methods in terms of their results—cancellous bone density at dental implant sites. Dental implants are replacements for missing teeth. Therefore, the survival rate of the implants must be improved. Bone quality, which is generally used as the basis for the quantitative evaluation of cancellous bone density, is strongly associated with osseointegration [31]. Although CT and dental CBCT have been widely used to measure cancellous bone density at dental implant sites [15–25, 30, 32], a standard approach for density measurement remains to be established. Furthermore, whether different ROI methods affect the measurement results remains to be clarified. To the best of our knowledge, this study is the first to investigate the correlations between different ROI methods used for preoperatively measuring bone density at dental implant sites by using dental CBCT images. Significant correlations were identified between the results of the three ROI methods assessed in this study. Thus, the choice of approach may not influence the results of cancellous bone density measurement. The density value obtained using the surrounding cylinder method was the highest.

The implant survival rate is strongly correlated with bone quantity, and cancellous bone density is a crucial parameter for the image-based evaluation of bone quantity. Freiberg et al. [33] investigated the correlation between bone density and implant survival rate in 4641 patients who received dental implants; unfortunately, dental implant failure was noted in 69 patients. The implant site was the posterior maxilla in most failed cases (49/69), where the bone density and quality was relatively poor. In a systematic review study published in 2017, the effects of implant site bone quality and quantity on implant failure rate were explored [34]. In the reviewed studies (*n* = 94) comparing bone quantity and implant failure rate at dental implant sites, the failure rates at sites with quality levels 1, 2, 3, and 4 were 3.38% (81/2359), 3.13% (486/15,544), 4.27% (722/16,920), and 8.06% (354/4293), respectively. The study also reviewed 55 studies on the correlation between bone quantity and implant failure rate. The findings are consistent with the literature on bone quantity. The failure rates at three implant sites with satisfactory bone quantity were approximately 3.98%, 3.75%, and 4.74%, whereas the rates at two implant sites with the poorest bone quantity were 8.74% and 18.98%. Cancellous bone is a porous structure comprising trabecular bone tissues. Compared with bones with lower density, cancellous bone has higher density and can thus offer a relatively large bone–implant contact area for the placement of a dental implant. A higher 3D bone–implant contact percentage indicates a tighter connection between the bone and the dental implant, which increases the initial stability of the implant [35]. A higher level of initial stability ensures more satisfactory osseointegration conditions, higher stability of dental implants, and a lower chance of failure. On the basis of the aforementioned observations, the bone density at dental implant sites is correlated with the rate of implant survival. A lower level of cancellous bone density and a poorer quantity of bone is more likely to result in dental implant failure. Conversely, a higher level of cancellous bone density and satisfactory quantity of bone is more likely to increase the rate of implant survival.

In the present study, the unit of radiographic density measured using CBCT images was GV, and that of radiographic density measured using CT images was HU ($HU = 1000 \times \frac{{\mu - \mu \,{\text{water}}}}{{\mu \,{\text{water}} - \mu \,{\text{air}}}}$). HU is the value obtained by calibrating the linear attenuation coefficients of water and air; therefore, HU can directly serve as a reference value for measuring the actual density of an object. However, GV obtained by assessing CBCT images may be influenced by multiple factors (e.g., machine brand and model). Hence, some researchers believe that the CBCT image–based measurement of bone density is inaccurate. Silva et al. [36] used CBCT and multislice CT (MSCT) images to assess 40 potential implant sites and found significant differences between the measurement results obtained using CBCT and MSCT images. Therefore, they reported that the bone density value obtained using CBCT images was unreliable because it was higher than that obtained using MSCT images. Varshowsaz et al. [37] reached a similar conclusion. They reported that CBCT image–based measurement does not produce accurate results; hence, such bone density measurement approach is unreliable. Nonetheless, they indicated that the measurement results are not affected by the thickness, acquisition parameters, or locations of the measured objects. In many studies, although the density measurement results obtained using CBCT images were not the absolute values like using CT images, the two approaches were highly positively correlated in terms of their results [38, 39]. Parsa et al. [38] used CT and micro-CT images as the standards to evaluate the results obtained using CBCT images; the GV value obtained using CBCT images, the ratio of trabecular bone volume to total volume obtained using micro-CT, and the HU values were highly correlated, which indicated the potential of CBCT for the evaluation of bone density at dental implant sites. Furthermore, the applicability of CBCT in the preoperative evaluation of dental implant surgery according to the accuracy of bone density estimated using CBCT has been investigated [39]; CBCT images were useful for measuring the density of jawbones and served as an effective evaluation tool before dental implant surgery. Genisa et al. acknowledged that bone density assessment in CBCT relies on measuring attenuation using Hounsfield units (HU), which is relative to the water attenuation coefficient. The study revealed a logarithmic relationship between CBCT Hounsfield units and bone density, contrasting with a linear correlation [29]. CBCT measurements can be affected by various measurement parameters and equipment differences among manufacturers. To minimize these influences on experimental outcomes, this study employed a BMD calibration phantom. The measured values were then converted into quantitative BMD values using this standardized approach.

In the literature on CT- and CBCT-based evaluation of bone density before dental implant surgery [15–19, 21–24, 30, 40] (Table 4), multiple measurement approaches and ROI methods have been adopted, which could roughly be divided into 2D and 3D categories. The ROI in 2D images was mainly a rectangular area on a single slice [17, 18, 30]. This area was used to simulate a dental implant site, and the mean radiographic density within the area was measured. By contrast, the mean density within a 3D area was measured using a multi-slice section. The two common ROI methods in the 3D category were inside cylinder [19, 23] and surrounding cylinder [15, 16, 21, 22]. In the inside cylinder ROI method, the area within the virtual cylinder simulating a dental implant was measured; conversely, in the surrounding cylinder method, the peripheral area of the virtual dental implant—the area in actual contact with the implant—was measured. Research conducted to measure bone density at potential implant sites has indicated that the density of the mandible was higher than that of the maxilla, regardless of the ROI method used. In addition, the density of the anterior region was likely to be higher than that of the posterior region. The bone density of the anterior mandible and posterior maxilla was the highest and lowest, respectively. The results of the present study indicated that bone density was the highest at the anterior mandible, followed by at anterior maxilla, posterior mandible, and posterior maxilla; this finding is consistent with the literature. However, the absolute values we obtained varied substantially from those reported in the literature because of the different ROI methods we adopted in this study.

Chougule et al. previously compiled a reference table of Hounsfield Unit (HU) values for various anatomical regions in the human body. In adults, the HU values for cortical bone were found to range between 662 and 1988 HU, while cancellous bone fell within the range of 148 to 661 HU [41]. However, the CT-based measurement results reported by Norton and Gamble [15] and Turkylimaz et al. [17, 30] were markedly higher than the CT- measurement results reported by other studies. This might be because Norton and Gamble and Turkylimaz et al. began the measurement from the apex of the crestal bone (including the crestal cortical bone area), whereas the others mostly measured the cancellous bone only. The HU value obtained using CT images can reflect the actual density of a scanned object. Therefore, we compared the studies in which CT was performed for measurement. The results revealed that the bone density value obtained using the surrounding cylinder method [16] was the highest, followed by those obtained using the cylinder [19] and then rectangle [18] methods.

In the present study, the bone density measured using the surrounding cylinder method was the highest. This might be because the range of measurement in the surrounding cylinder method was the largest and could easily include cortical bones, which increased the mean value and resulted in the overestimation of cancellous bone density. Although the surrounding cylinder is the area in actual contact with a dental implant, cortical bones should be avoided during bone density measurement. A study [42] reported that cortical and cancellous bones differentially affect the stability of dental implants. Cortical bone exhibits a stronger correlation with the initial stability of dental implants, whereas cancellous bone exhibits a stronger correlation with subsequent osseointegration. Therefore, the two bones should be assessed separately in the evaluation of bone density before dental implant surgery. Thus, when using the surrounding cylinder method, caution must be exercised to avoid the overestimation of cancellous bone density because of the inclusion of the cortical bone.

**Table 4:** Various cancellous bone density ROI methods used in relevant studies

Author and year	Ethnic group	Measurement Method	Scanning Device	Unit	Resolution	Scanning parameter	Anterior Mandible	Anterior Maxilla	Posterior Mandible	Posterior Maxilla
Norton and Gamble (2001) [15]	UK	Surrounding cylinder	CT	Hounsfield unit (HU)	--	-- (GE ProSpeed)	970 ± 269	696 ± 244	669 ± 248	364 ± 227
Shapurian et al. (2006) [16]	USA	Surrounding cylinder	CT	Hounsfield unit (HU)	slice thickness 1 mm	-- (GE Hi-Speed)	559 ± 208	517 ± 177	321 ± 119	333 ± 119
Turkyilmaz et al. (2007) [17]	USA	Rectangle	CT	Hounsfield unit (HU)	slice thickness 1 mm slice intervals 1 mm	130kVp, 83 mA (Siemens AR-SP 40)	945 ± 207	716 ± 190	445 ± 122	674 ± 227
De Oliveira et al. (2008) [18]	Sweden	Rectangle	CT	Hounsfield unit (HU)	1-mm-thick slices 250-mm FOV 512 * 512 matrix	140kVp, 200-400mAs (Elscint Twin II helical scanner)	383 ± 243	370 ± 176	255 ± 184	306 ± 187
Turkyilmaz and McGlumphy [30] (2008)	USA	Rectangle	CT	Hounsfield unit (HU)	slice thickness 1 mm slice intervals 1 mm	130kVp, 83 mA (Siemens AP-SR 40)	846 ± 234	591 ± 176	526 ± 107	403 ± 95
Fuh et al. (2010) [19]	Taiwan	Cylinder	CT	Hounsfield unit (HU)	240 mm FOV 512*512 pixels	120kVp, 300-400mAs (General Electric LightSpeed)	530 ± 161	516 ± 132	332 ± 136	359 ± 150
Hiasa et al. (2011) [40]	Japan	Combination of inside and outside cylinder	CT	Hounsfield unit (HU)	slice thickness 0.5 mm slice increment 0.3 mm	135kVp, 150 mA (Toshiba TSX-101 A)	844 ± 242	641 ± 173	628 ± 209	486 ± 199
Salimov et al. (2014) [21]	Turkey	Surrounding cylinder	CBCT	Density value	0.2 mm	120kVp, 5 mA (Imtec Iluma)	928 ± 220	732 ± 163	669 ± 194	459 ± 108
Hao et al. (2014) [22]	China	Surrounding cylinder	CBCT	Hounsfield unit (HU)	0.2 mm	90kVp, 14 mA (Planmeca ProMax 3D)	680 ± 142	460 ± 136	394 ± 128	230 ± 144
David et al. (2014) [23]	Romania	Cylinder	CBCT	Hounsfield unit (HU)	0.2 mm	84kVp, 14 mA (Planmeca ProMax 3D)	F:514 ± 243 M:521 ± 247	F:354 ± 212 M:473 ± 220	F:234 ± 206 M:389 ± 208	F:193 ± 176 M:250 ± 193
Felicori et al. (2015) [24]	Brazil	Cylinder and Surrounding cylinder	CBCT	Grayscale	0.2 mm	120kVp (i-CAT TC)	562 ± 2.81	531 ± 2.77	394 ± 2.8	503 ± 2.82
This study	Taiwan	Rectangle	CBCT	Grayscale value (GV) / BMD(g/cm^3^)	0.2 mm	96kVp, 12.5 mA (Planmeca ProMax 3D)	723 ± 227 / 523 ± 100	542 ± 220 / 369 ± 94	533 ± 212 / 361 ± 87	386 ± 201 / 236 ± 78
	Taiwan	Cylinder	CBCT	Grayscale value (GV) / BMD(g/cm^3^)	0.2 mm	96kVp, 12.5 mA (Planmeca ProMax 3D)	710 ± 227 / 512 ± 100	541 ± 215 / 368 ± 90	530 ± 204 / 359 ± 80	384 ± 198 / 234 ± 75
	Taiwan	Surrounding cylinder	CBCT	Grayscale value (GV) / BMD(g/cm^3^)	0.2 mm	96kVp, 12.5 mA (Planmeca ProMax 3D)	744 ± 225 / 541 ± 98	581 ± 214 / 402 ± 89	562 ± 195 / 386 ± 73	406 ± 191 / 253 ± 69

The correlation between the rectangle and cylinder ROI methods was the strongest. This is because although measurement was performed using the rectangle method on only a single slice, the area was a layer in the cylinder. By contrast, the correlation between the rectangle and surrounding cylinder methods was the weakest. This might be because of two reasons. First, compared with the cylinder method, where the measurement was performed using a multislice section, in the rectangle method, the measurement was performed using a single slice; hence, a mean density value was obtained. The measurement results of the rectangle method were more likely to vary because of the slice selected. Second, because in the surrounding cylinder method, the measurement might have included the cortical bone area, the results were likely to be inconsistent and overestimated. Nevertheless, the pair-wise correlations between the three ROI methods were strong, which implies that the choice of method may not influence the measurement results. We further divided the jawbone into four regions to investigate the aforementioned correlations in different regions of the jawbone. In all four regions, the measurement results obtained using the three ROI methods exhibited significantly strong correlations. The correlations between the surrounding cylinder and the other two ROI methods in the anterior region (anterior maxilla: *r* = 0.946 and 0.966, respectively; anterior mandible: *r* = 0.940 and 0.955, respectively) were lower than those in the posterior region (posterior maxilla: *r* = 0.961 and 0.976, respectively; posterior mandible: *r* = 0.962 and 0.971, respectively). As mentioned, the mean density obtained using the surrounding cylinder method was the highest because the cortical bone was also measured in this ROI. The results of the pair-wise correlation analysis further supported the trend in the anterior region. This might be because jawbones are narrower in the anterior region than in the posterior region. Because of the width limitation of the jawbone in the anterior region, in clinical practice, the diameter of dental implants placed in the anterior region is considerably smaller than that of the dental implants placed in the posterior region.

The ANOVA results in this study revealed that the measurement results of the surrounding cylinder ROI method were significantly higher than those of the other two methods. No significant difference was observed between the rectangle and cylinder ROI methods in terms of the measurement results obtained in the four jawbone regions. This might be because the rectangle was the central slice of the cylinder. Although larger errors might occur in single-slice measurement than in multislice measurement, the differences between the two ROI methods may not be large. Furthermore, except for the measurement results of the surrounding cylinder and rectangle ROI methods obtained at the anterior mandible, the bone density obtained using the surrounding cylinder method was significantly higher than that obtained using the other two ROI methods. Hiasa et al. [40] measured cancellous bone density at different implant sites before dental implant surgery. They measured bone density inside and outside the simulated implant. The results indicated that in female patients, the density measured outside the simulated implant (619.6 ± 208.8 HU) was significantly higher than that measured inside it (474.2 ± 230.4 HU). Likewise, in male patients, the density measured outside the simulated implant was likely to be higher than that measured inside it; however, the differences were nonsignificant. Arisan et al. [43] investigated the correlation between the radiographic density measured using CT and CBCT images and the stability of dental implants. The cylinder and surrounding cylinder ROI methods were adopted for bone density measurement. For both CT and CBCT images, the density measured using the surrounding cylinder method was significantly higher than that measured using the cylinder method. In the present study, the use of the rectangle and surrounding cylinder methods led to no significant differences in the measurement results obtained in the anterior mandible region. This might be because the jawbone is relatively narrow in this region, and the surrounding cylinder may include the cortical bone. Because the rectangle method is a 2D approach, the results might have varied depending on the slice selected. The anterior mandible region had fewer samples, which might have resulted in larger errors in the measurement results obtained using the two ROI methods and the nonsignificant differences between their results.

To avoid overestimation when using the surrounding cylinder method, measurement in the anterior region should be carefully performed. To avoid errors when using the rectangle method, caution should be exercised while selecting slices for measurement. Despite the fact that the area measured using the cylinder method is not the area in actual contact with dental implants, the cancellous bone density is less likely to be overestimated because of the inclusion of cortical bone, and the overall density may be effectively measured because 3D imaging is performed in this method. Therefore, the cylinder ROI method appears to be more suitable than the other two method.

This study has some limitations. First, a rectangle and cylinder were used as virtual dental implants; however, not all implants have straight structures; some have a tapered structure. Nevertheless, the effect is likely to be small and thus would not have influenced our findings. Second, because the jawbone is relatively narrow in the anterior region, the cortical bone is also measured in the surrounding cylinder method; thus, the density value obtained using this method was higher than those obtained using the other two methods. Finally, we could not analyze the consistency between the obtained results and subsequent implant survival and stability. Hence, the most accurate ROI method could not be identified. Nonetheless, we found that the three ROI methods were strongly correlated.

## Conclusions

Based on the CBCT equipment and scanning parameters settings employed in this study, the three ROI methods appear to be strongly correlated in terms of measurement results; the choice of ROI method may not influence the estimated bone density results. Nevertheless, in this study, bone density measured using the surrounding cylinder method was the highest, particularly in the anterior region.

## Data Availability

No datasets were generated or analysed during the current study.
